# Neurohypophysial Receptor Gene Expression by Thymic T Cell Subsets and Thymic T Cell Lymphoma Cell Lines

**DOI:** 10.1080/10446670410001670481

**Published:** 2004-03

**Authors:** I. Hansenne, G. Rasier, Ch. Charlet-renard, M.-P. Defresne, R. Greimers, C. Breton, J.-J. Legros, V. Geenen, H. Martens

**Affiliations:** 1 Center of Immunology University of Liege Institute of Pathology CHU-B23 Liege-Sart Tilman B-4000 Belgium; 2 Center of Cancerology University of Liege Institute of Pathology CHU-B23 Liege-Sart Tilman B-4000 Belgium; 3 Endocrinology of Annelidae Laboratory University of Lille I Villeneuve d'Ascq F-59655 France

## Abstract

Abstract Neurohypophysial oxytocin (*OT*) and vasopressin (*VP*) genes are transcribed in thymic epithelium, while immature T lymphocytes express functional neurohypophysial receptors. Neurohypophysial receptors belong to the G protein-linked seven-transmembrane receptor superfamily and are encoded by four distinct genes, *OTR*, *V1R*, *V2R* and *V3R*. The objective of this study was to identify the nature of neurohypophysial receptor in thymic T cell subsets purified by immunomagnetic selection, as well as in murine thymic lymphoma cell lines RL12-NP and BW5147. *OTR* is transcribed in all thymic T cell subsets and T cell lines, while *V3R* transcription is restricted to CD4^+^ CD8^+^ and CD8^+^ thymic cells. Neither *V1R* nor *V2R* transcripts are detected in any kind of T cells. The OTR protein was identified by immunocytochemistry on thymocytes freshly isolated from C57BL/6 mice. In murine fetal thymic organ cultures, a specific OTR antagonist does not modify the percentage of T cell subsets, but increases late T cell apoptosis further evidencing the involvement of OT/OTR signaling in the control of T cell proliferation and survival. According to these data, *OTR* and *V3R* are differentially expressed during T cell ontogeny. Moreover, the restriction of *OTR* transcription to T cell lines derived from thymic lymphomas may be important in the context of T cell leukemia pathogenesis and treatment.


*Clinical & Developmental Immunology*, March 2004, Vol. 11 (1), pp. 45–51

Taylor & Francis healthsciences

### Neurohypophysial Receptor Gene Expression by Thymic T Cell Subsets and Thymic T Cell Lymphoma Cell Lines

I. HANSENNE[a,*], G. RASIER[a,*], CH. CHARLET-RENARD[a], M.-P. DEFRESNE[b], R. GREIMERS[b], C. BRETON[c], J.-J. LEGROS[a], V. GEENEN[a,†] and H. MARTENS[a]

[a]*Center of Immunology;* [b]*Center of Cancerology, University of Liege, Institute of Pathology CHU-B23, B-4000, Liege-Sart Tilman, Belgium;* [c]*Endocrinology of Annelidae Laboratory, University of Lille I, F-59655, Villeneuve d'Ascq, France*

Neurohypophysial oxytocin (*OT*) and vasopressin (*VP*) genes are transcribed in thymic epithelium, while immature T lymphocytes express functional neurohypophysial receptors. Neurohypophysial receptors belong to the G protein-linked seven-transmembrane receptor superfamily and are encoded by four distinct genes, *OTR*, *V1R*, *V2R* and *V3R*. The objective of this study was to identify the nature of neurohypophysial receptor in thymic T cell subsets purified by immunomagnetic selection, as well as in murine thymic lymphoma cell lines RL12-NP and BW5147. *OTR* is transcribed in all thymic T cell subsets and T cell lines, while *V3R* transcription is restricted to $CD4^+CD8^+$ and $CD8^+$ thymic cells. Neither *V1R* nor *V2R* transcripts are detected in any kind of T cells. The OTR protein was identified by immunocytochemistry on thymocytes freshly isolated from C57BL/6 mice. In murine fetal thymic organ cultures, a specific OTR antagonist does not modify the percentage of T cell subsets, but increases late T cell apoptosis further evidencing the involvement of OT/OTR signaling in the control of T cell proliferation and survival. According to these data, *OTR* and *V3R* are differentially expressed during T cell ontogeny. Moreover, the restriction of *OTR* transcription to T cell lines derived from thymic lymphomas may be important in the context of T cell leukemia pathogenesis and treatment.

*Keywords*: Neurohypohysial receptor; Thymus; T cells; Lymphoma; Leukemia

### INTRODUCTION

A repertoire of neuroendocrine-related genes are expressed within the thymus parenchyme of different species (Martens *et al.*, 1996). Thymic neuroendocrine-related genes are highly conserved throughout evolution of their family and one dominant member is expressed in the thymus network: insulin-like growth factor 2 (*IGF2*) for the insulin family, neurokinin A (*NKA*) for tachykinins (Ericsson *et al.*, 1990), and neurotensin (*NT*) for neuromedins (Vanneste *et al.*, 1997). Thymic precursors encoded by these genes exert a dual role in T cell differentiation according to their behavior either as cryptocrine/juxtacrine ligands or as neuroendocrine self-antigens (Geenen *et al.*, 1999; Geenen *et al.*, in press). With regard to the neurohypophysial gene family, both oxytocin (*OT*) and vasopressin (*VP*) genes are expressed in thymic epithelial and nurse cells (TEC/TNC) from different species. At the peptide level however, thymic OT concentration is much higher than VP (Robert *et al.*, 1992; Geenen *et al.*, 1998). During mouse ontogeny, *OT* and *VP* expression starts in thymic epithelium on embryonic day E13, while neurohypophysial transcripts are evidenced in the brain only on E15 (submitted for publication). Specific OT and VP binding sites have been evidenced in the rat thymus (Elands *et al.*, 1990), as well as on a murine $CD4^-CD8^-$ T cell line (RL12-NP) derived from X ray-induced thymic lymphoma in C57BL/Ka mice (Martens *et al.*, 1992). These binding sites function as true receptors and are able to transduce neurohypophysial ligands into phosphoinositide turnover followed by proliferation effects (Martens *et al.*, 1992). More specifically, neurohypophysial peptides, and OT in particular, markedly stimulate the phosphorylation of focal adhesion kinases in RL12-NP cells (Martens *et al.*, 1998).

Distinct genes (*OTR*, *V1R*, *V2R* and *V3R*) encode four neurohypophysial peptide receptors which all belong to the superfamily of G-coupled seven-transmembrane proteins. *OTR* is primarily expressed in uterus myometrium, *V1R* in vascular smooth muscle and endothelium as well as in liver epithelial cells, *V2R* in the epithelium of kidney water collecting tubules, and *V3R* in anterior pituitary corticotroph cells (Gainer and Wray, 1994). OTR, V1R and V3R are coupled to protein $G_{q/11}$ and activate phospholipase C pathway (Thibonnier *et al.*, 1993), while V2R is coupled to protein $G_s$ and activates adenylate cyclase pathway (Barberis and Tribollet, 1996).

*Both authors contributed equally to this work.
†Corresponding author. Tel.: +32-4-366-25-50. Fax: +32-4-366-29-77. E-mail: vgeenen@ulg.ac.be

ISSN 1740-2522 print/ISSN 1740-2530 online © 2004 Taylor & Francis Ltd
DOI: 10.1080/10446670410001670481

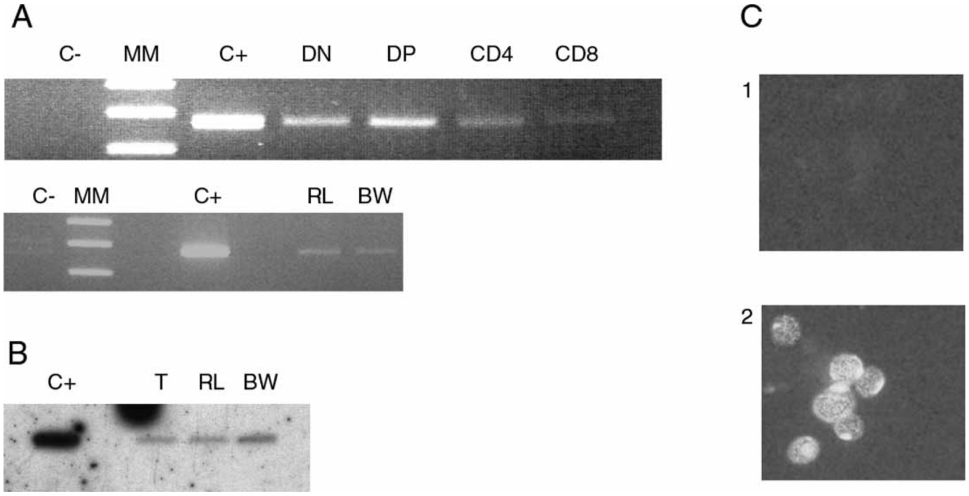

FIGURE 1 *OTR* expression. (A) RT-PCR in murine T cell subsets and lymphoid T cell lines; C − : negative control; MM: molecular mass marker; C + : positive control; DN: CD4$^-$CD8$^-$; DP: CD4$^+$CD8$^+$; CD4: CD4$^+$; CD8: CD8$^+$; RL: CD4$^-$CD8$^-$ lymphoid RL12-NP T cell line; BW: CD4$^-$CD8$^-$ lymphoid BW5147 T cell line. (B) Southern-blotting in thymocytes (T) and lymphoid T cell lines (RL, BW). (C) Immunocytochemistry on freshly isolated thymocytes. 1: negative control; 2: anti-OTR antibody. Magnification, × 400.

This study aimed to determine the nature and distribution of *OTR*, *V1R*, *V2R* and *V3R* expressed by murine thymic T cell subpopulations and by thymic lymphoma-derived T cell lines RL12-NP and BW5147. In addition, a preliminary insight into the functional role played by the thymic neurohypophysial axis in T cell development was investigated through the use of fetal thymic organ cultures (FTOC) (Jenkinson and Anderson, 1994).

### RESULTS

### Neurohypophysial Receptor Expression

#### *OTR Expression*

RT-PCR with *OTR* specific primers revealed the presence of a 273-bp product in the mouse uterus as positive control. A same sized RT-PCR product was detected in all thymic T cell subsets, and in RL12-NP and BW5147 lines (Fig. 1A). Southern blotting of the PCR product and hybridization with a specific probe confirmed the presence of *OTR* transcripts in thymocytes (Fig. 1B). Further sequencing of the 237-bp product amplified from RL2-NP cells showed a 97.1% homology with murine *OTR* sequence. Thymocytes freshly isolated from C57BL/6 mice were also positively stained with a specific Ab to OTR (Fig. 1C).

#### *V3R Expression*

RT-PCR with *V3R* primers identified a 254-bp product in mouse anterior pituitary as positive control. A same sized product was similarly detected in thymic CD4$^+$CD8$^+$ and CD8$^+$ T cells, but neither in CD4$^-$CD8$^-$ nor in CD4$^+$ T cells (Fig. 2A). No amplified product was detected in RL12-NP and BW5147 lines (not shown). Southern blotting and hybridization with a specific probe confirmed *V3R* expression in CD4$^+$CD8$^+$ and CD8$^+$ thymic T cells (Fig. 2B).

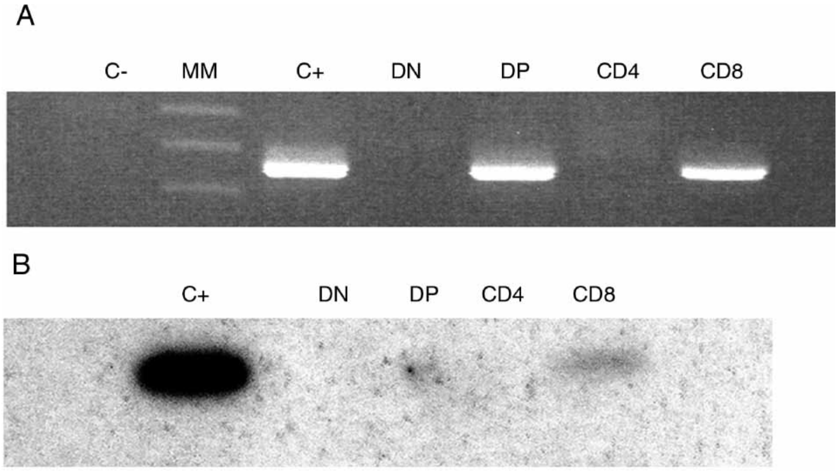

FIGURE 2 *V3R* expression. (A) RT-PCR in murine T cell subsets. (B) Southern blotting in murine T cell subsets. See Fig. 1 legend for abbreviations.

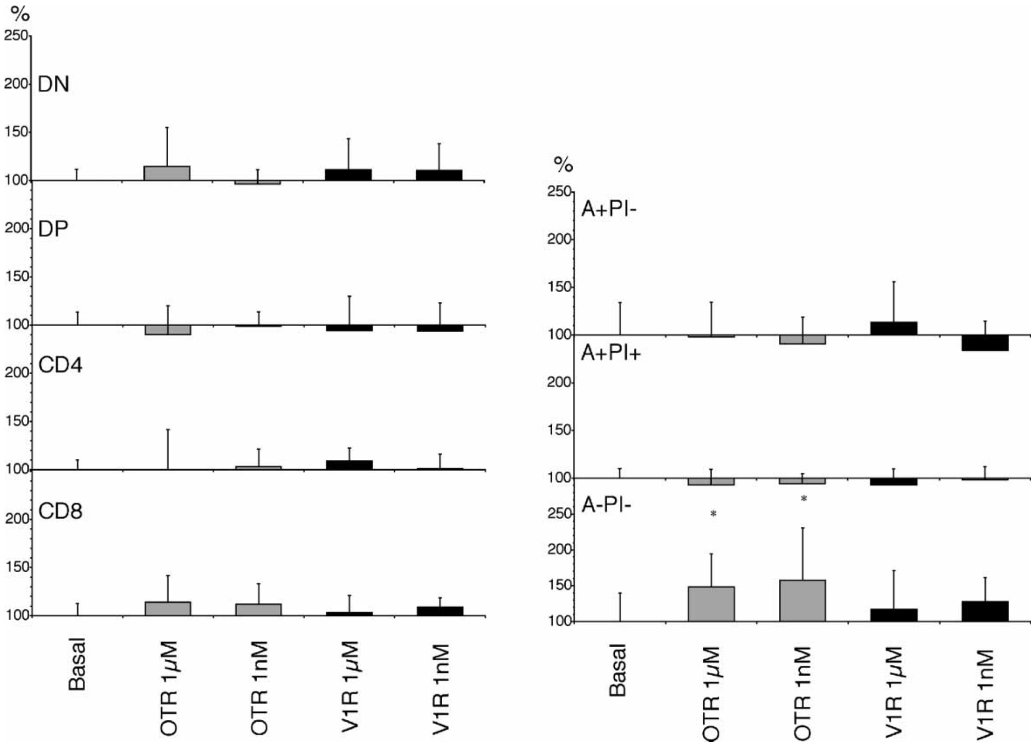

FIGURE 3 (A) Percentage of T cell subsets collected from FTOC after treatment with OTR and V1R antagonists. (B) Staining with apoptosis markers of isolated thymocytes from FTOC after treatment by OTR and V1R antagonists; A + PI − : early apoptosis cells; A + PI + : late apoptosis cells; A − PI − : living cells. Each column represents percentage (m ± ds) of variation by comparison with basal conditions (100%). *$p < 0.05$; **$p < 0.01$.

### *V1R and V2R Expression*

RT-PCR with *V1R* and *V2R* primers revealed the presence of 409 and 437-bp products in liver and kidney, respectively, but failed to amplify any product in thymic T cells and T cell lines. Hybridization with specific probes confirmed the absence of *V1R* and *V2R* transcripts in all T cell samples (data not shown).

### Effects of Neurohypophysial Receptor Antagonists on T Cell Differentiation and Survival in FTOC

From day 0 (corresponding to E14) to day 6 of FTOC, RT-PCR with *OT* and *VP* specific forward and reverse primers revealed 211 and 188-bp products, respectively, which hybridized with specific OT and VP oligonucleotide probes after membrane transfer. In FTOC treated with specific OTR and V1R antagonists at $10^{-6}$ and $10^{-9}$ M, no significant effect could be observed on T cell differentiation. However, the OTR antagonist ($10^{-6}$ and $10^{-9}$ M)—but not the V1R antagonist—induced a significant increase of late apoptosis parameters (Fig. 3).

### DISCUSSION

As suggested by previous studies (Torres and Johnson, 1988; Elands *et al.*, 1990; Martens *et al.*, 1992), T lymphocytes express neurohypophysial peptide receptors, but the precise identity of receptor subtypes and positive T cells still had to be fully characterized. The present study addressed this question by investigating the nature of neurohypophysial receptor expressed by highly purified thymic T cells and by thymic lymphoma-derived T cell lines RL12-NP and BW5147. As clearly shown, *OTR* is transcribed in all MACS-purified thymic T cell subsets ($CD4^-CD8^-$, $CD4^+CD8^+$, $CD4^+$ and $CD8^+$), as well as in RL12-NP and BW5147 T cell lines. *OTR* transcription leads to membrane expression of the OTR protein in freshly isolated thymocytes. These data confirm at the molecular level those previously obtained by autoradiography and by classic radio-ligand binding analyses (Elands *et al.*, 1990; Martens *et al.*, 1992).

RT-PCR with *V3R* primers identified in $CD4^+CD8^+$ and $CD8^+$ cells a product with the same size as the anterior pituitary positive control. Southern blotting and hybridization with a specific probe confirmed that *V3R* is expressed by these T cell subpopulations. This result indicates that V3R might be the other neurohypophysial receptor expressed by T cells as already suggested in a previous study (Martens *et al.*, 1992). Our observation also explains the positive hybridization with *V3R* cDNA in northern blots from human thymus extracts (Sugimoto *et al.*, 1994). Nevertheless, we cannot definitively exclude that other cell types might also express *V3R* in the thymus cellular network.

*V1R* expression could not be documented in any of the T cell types investigated. Since vascular endothelial cells express this receptor (Barberis and Tribollet, 1996; Zingg, 1996), this negative result indirectly confirms that our purification procedure is not contaminated by endothelial cells. *V2R* expression was neither observed in any type of T cells and this concords with our experience about the absence of adenylate cyclase activation by neurohypophysial-related peptides in immature or mature T cells. Previously, a radiobinding study had suggested that, in the immune system, only macrophages express the *V2R* type of neurohypophysial receptor (Block *et al.*, 1981).

The functional role of thymic neurohypophysial-related peptides must be discussed according to the behavior of the final peptide product processed in TEC following *OT* and *VP* transcription. This study characterizes the parameters involved in the signaling between neurohypophysial ligands and receptors, and these may be currently defined as follows. At the level of the neurohypophysial ligands, both OT and VP peptides are synthesized in thymic epithelial cells (TEC), with a dominance of OT. At the level of the receptors, *OTR* is expressed at all main stages of T cell differentiation, while *V3R* expression is restricted to $CD4^+CD8^+$ and $CD8^+$ T cells. Thus, a different pattern of neurohypophysial receptor expression occurs along with the process of T cell differentiation. Similarly to what has been shown for IGF type 2 receptors (CD222) (Mason, 2000), it may be expected that OTR and V3R also will be soon identified as new cluster differentiation (CD) markers. Ancient studies have reported that neurohypophysial ligands increase thymocyte growth (Whitfield *et al.*, 1969; Martens *et al.*, 1992) and the level of glucose oxydation in the thymus (Goren *et al.*, 1984). More recently, the binding of neurohypophysial ligands, particularly OT, was shown to increase the level of inositol triphosphate and to phosphorylate focal adhesion-related kinases (FAK) in immature T cells (Martens *et al.*, 1992, 1998). The implication of these kinases in T cell differentiation has also been described by other authors (Kanazawa *et al.*, 1996). In this study, the FTOC model was used to gain a first insight into the role of the thymic neurohypophysial axis in T cell development. Endogenous *OT* and *VP* transcription was controlled throughout FTOC duration. FTOC treatment with a specific OTR antagonist ($10^{-6}$ and $10^{-9}$ M) induced a significant increase in late apoptosis parameters, but did not modify the percentage of T cell subsets. A V1R antagonist did not exert any significant influence, in accordance with the absence of *V1R* expression by thymic T cell subsets. These data further confirm that an intrathymic OT/OTR signaling is implicated in the general process of T cell development and survival. Since this pathway also controls FAK phosphorylation in $CD4^-CD8^-$ RL12-NP cells, thymic OT/OTR signaling could play a significant role in the promotion of immunological synapses between TEC and thymocytes during T cell differentiation.

Finally, it should be stressed that the investigation of neurohypophysial receptor expression by immature $CD4^-CD8^-$ T cells lines derived from murine thymic lymphomas showed that *OTR* is the only neurohypophysial receptor expressed by those cells. With regard to the mitogenic and FAK-promoting activity of thymic OT, as well as proapoptotic properties of the OTR antagonist, this finding suggests that the use of OTR antagonist might be considered in the future therapy of T cell lymphomas/leukemia's.

### MATERIALS AND METHODS

### Cell Lines and Reagents

Immature T cell lines are RL12-NP derived from X ray-induced thymic lymphoid tumor in C57BL/Ka mice (Lieberman *et al.*, 1979) and BW5147, established from radio-induced thymic lymphoma in female C57BL/6 (Vink *et al.*, 1993). These T cell lines exhibit an immature $CD4^-CD8^-$ phenotype. Polyclonal Ab to OTR was kindly gifted by Paola Cassoni (University of Torino, Italy) (Bussolati *et al.*, 1996). The OTR antagonist des-Gly-$NH_2d(CH_2)_2$[D-$Tyr^2$, $Thr^4$]OVT), and the V1R antagonist $d(CH_2)_5$-Tyr(Me)AVP were kindly provided by Maurice Manning (Medical College of Ohio, Toledo, OH).

### Staining and Immunomagnetic Separation (MACS)

Thymic lobes were surgically removed from adult female C57BL/6 mice; they were cut in small pieces, filtered and washed in DPBS buffer. Thymocyte suspension was incubated with a mAb cocktail prepared for negative selection of CD4 and CD8 T cells (StemCell Technologies). After washing in DPBS buffer, an anti-biotin tetrameric Ab complex mix (StemCell Technologies) was added. The last incubation was performed with a magnetic colloid (StemCell Technologies). Separation was realized with a MACS column (Miltenyi Biotec).

### FTOC

Fetal thymic lobes were removed from Balb/c murine embryos on E14, the day 0 being the day of vaginal plug observation. Thymic lobes were cultured on a Nucleopore filter (Corning), put on a sterile sponge (Gelfoam), as previously described (Kecha *et al.*, 2000). Culture medium was Iscove filtered Medium modified by the Dulbecco's Method (IMDM, Cambrex), enriched with L-glutamin (2 mM), HEPES (10 mM), non essential amino acids (1%), natrium pyruvate (1 mM), penicillin (50 IU/ml), streptomycin (50 ng/ml) and 10% fetal bovine serum (Life Technologies).

**Flow Cytometry Analyses and Sorting**

Double staining was achieved on FTOC T cells with anti-CD4 coupled to phycoerythrin (PE) (clone GK 1.5), and anti-CD8 coupled to fluorescein (FITC) (clone 53-6.7). Apoptosis markers were FITC-annexin V (Biosource) and propidium iodide (PI) (Sigma). Quadruple stainings were realized with anti-CD4 Ab (PE), biotinylated (Biot) anti-CD8 Ab followed by Per-CP-coupled streptavidin (PerCP) (Becton-Dickinson), FITC-Annexin V (Biosource) and PI (Sigma).

**Total RNA Extraction**

Total RNA from murine uterus, liver, kidney and anterior pituitary was extracted with TriPure Isolation Reagent (Boerhinger Mannheim). Total RNA from thymocytes, cell lines and fetal thymic lobes (at different days of culture) was extracted with RNeasy Mini Kit (Qiagen). They were treated (15 min, 37°C) with DNase I (5 IU/reaction) in presence of RNase inhibitor (2 IU/μl). Reaction was ended by enzyme inactivation (8 min, 85°C). Then, 0.1vol NaAc (3 M) and 2.5vol ethanol (100%) were added and RNA was precipitated (12 h, −20°C). After two washings with ethanol (70%), RNA was suspended in milli-Q quality water and quantified with Ribogreen RNA Quantitation Kit (Molecular Probes).

**RT-PCR**

Primers were designed from murine *OTR*, *V1R*, *V2R*, *V3R*, *OT* and *VP* sequences and selected in different exons in order to exclude genomic DNA from amplification. The sequences of the primers are:

*OTR* forward: 5′-CTGGGACGTCAATGCGCCCAA-AGAAG-3′ and

*OTR* reverse: 5′-CATGCCGAGGATGGTTGAGAAC-AGCTC-3′ (273-bp product)

*V1R* forward: 5′-TGGTCACGCCTTGTGTCAGCAG-CGTGA-3′ and

*V1R* reverse: 5′-GATTTAGGTGAATCCTTCCACGT-CCCA-3′ (409-bp product)

*V2R* forward: 5′-CCCTAGGCATTGCTGCCTGCC-3′ and

*V2R* reverse: 5′-GAAGCTGGCTGTGGCACAGGAC-TCATCTTG-3′ (437-bp product)

*V3R* forward: 5′-CGGGTCAGCAGCATCAGTACCA-TCTCC-3′ and

*V3R* reverse: 5′-GGAACGTGGCAACAGGTGGCTG-TTGA-3′ (254-bp product)

*OT* forward: 5′-ACCTCGGCCTGCTACATCCAGAA-3′ and

*OT* reverse: 5′-ACTGGCAGGGCGAAGGCAGGTA-3′ (211-bp product)

*VP* forward: 5′-GGCATCTGCTGCAGCGACGAGA-3′ and

*VP* reverse: 5′-TAGACCCGGGGCTTGGCAGAA-3′ (188-bp product)

RNA integrity and concentration were controlled by RT-PCR with primers derived from murine β-actin sequence (Pleau *et al.*, 1996):

*β-actin* forward: 5′-TAAAGACCTCTATGCCAACA-CAGT-3′ and

*β-actin* reverse: 5′-CACGATGGAGGGGCCGGACT-CATC-3′ (250-bp product).

Total RNA (50 ng) of each sample was reverse-transcribed (RT) using random hexamers (30 min, 42°C). This was followed by a denaturation step (5 min, 94°C) and a polymerase chain reaction (PCR) with 35 cycles (for *OTR*, *V1R*, *V2R*, *OT* and *VP*) or 37 cycles (for *V3R*) cycles. For each cycle, denaturation was 94°C for 45 s (*OT* and *VP*) or 1 min (*OTR*, *V1R*, *V2R* and *V3R*); primer hybridization was 1 min at 54°C (β-actin), 56°C (*OTR*, *V1R* and *V2R*), 64°C (*V3R*) and 45 s at 66°C (*OT* and *VP*); elongation phase was 1 min (*OTR*, *V1R*, *V2R*, *V3R*), or 1 min 30 s, (*OT* and *VP*) at 72°C. After amplification, an additional extension step at 72°C for 10 min was performed. RT-PCR products were analyzed on 2% agarose gel and stained with ethidium bromide (0.5 μg/ml).

Positive controls were RNA from adult Balb/c uterus (*OTR*), liver (*V1R*), kidney (*V2R*) and anterior pituitary (*V3R*). Milli-Q quality water was used as negative control.

**Southern Blotting**

Neurohypophysial receptor RT-PCR products were transferred for 60 min on Hybond-XL membrane (Amersham, Pharmacia Biotech), and neurohypophysial ligand RT-PCR products on Gene Screen Plus membrane (Dupont). Oligonucleotide probes were synthesized from gene sequence expected to be included in the amplified products:

*OTR*: 5′-GAGACGAGCATTAGCAAGAAAAGCAA-CTCCTCCACC-3′,

*V1R*: 5′-GAAATTGGTATCCCAGACTGACCACAT-CTGGACGATGAAG-3′,

*V2R*: 5′-CACCAGACTGGCATGTATCTCCCGGAA-GATAAGAACC-3′,

*V3R*: 5′-AAGCAATGTAGGCCAGCACAATGACA-AAGGTCATCTTC-3′,

*OT*: 5′-CCCCTGGGCGGCAAGAGGGCTGTGCTG-GACCTGGATATGC-3′,

*VP*: 5′-CACAGCTGGACGGCCCTGCTCGGGCGCT-GCTGCTAAGGCT-3′.

Oligonucleotides (50 ng) were labeled with [$\gamma^{32}$P] dATP (5 μl, 4500 Ci/mmol) using $T_4$ kinase (7.9 IU) (Pharmacia), then purified on BIO-RAD Micro Bio-spin Column P-30 Tris, RNase-Free.

Hybond XL membranes were prehybridized (2 h at 65°C) in Denhardt solution (5X), SSC (5X) and SDS (0.5%) followed by hybridization (12 h, 65°C) in the same solution with labeled probe (1,000,000cpm/ml). Membranes were successively washed in a SSC solution (2X) and SDS (0.1%) (5 min), SSC (1X) and SDS (0.1%) (15 min), SSC (0.1X) and SDS (0.1%) (10 min). Gene

Screen Plus membranes were prehybridized (2 h, 60°C) in SSC (5X), $Na_3PO_4$ (20 mM), Denhardt solution (50X), SDS (7%) and salmon sperm DNA (0.1 ng/ml); this was followed by hybridization (12 h, 60°C) in prehybridization solution + dextran sulfate (20%) with labeled OT (560,000 cpm/ml) or VP (820,000 cpm/ml) probe. Membranes were washed in SSC (5X) (30 min, 60°C) and in SSC (2X) (30 min, 60°C). cDNA was visualized with PhosphorImager after exposition (2 h) or by autoradiography (−70°C, 2 day exposition).

### Sequencing

RT-PCR products were subcloned in pCR2.1-TOPO plasmid (Invitrogen) and sequenced using ABI Prism BigDye Terminator Cycle Sequencing Ready Reaction Kit protocol (Perkin Elmer).

### Immunocytochemistry

Thymocytes were freshly isolated from C57BL/6 mice, transferred on slides by cytospin centrifugation and fixed acccording to Robert *et al.* (1991). OTR polyclonal Ab (1:50) was used as first Ab, and FITC goat anti-mouse Ig (Jackson, 1:50) was used in second step.

### Statistics

Results were expressed as mean (m) ± standard deviation (sd) of values after treatment vs basal conditions. Normality was tested by Kolmogorov–Smirnov test. Differences between groups were estimated by ANOVA and analyzed by multi-comparative Student-Newman–Keuls test.

### *Acknowledgements*

This work was supported by Fondation Leon Fredericq (Liege Medical School) and by Federation Belge contre le Cancer (Brussels). Vincent Geenen MD, PhD is Research Director of Belgian NFSR and Director of Liege Center of Immunology. Our gratitude is due to Dr Paola Cassoni (University of Torino, Italy) for OTR antibodies and to Pr. Maurice Manning (Medical College of Ohio, Toledo, OH) for neurohypophysial receptor antagonists.

### *References*

Barberis, C. and Tribollet, E. (1996) "Vasopressin and oxytocin receptors in the central nervous system", *Crit. Rev. Neurobiol.* **10**, 119–154.

Block, L.H., Locher, R., Tenschert, W., Siegenthaler, W., Hofmann, T., Mettler, R. and Vetter, W. (1981) "($^{125}$I)-8-L-arginine vasopressin binding to human mononuclear phagocytes", *J. Clin. Invest.* **68**, 374–381.

Bussolati, G., Cassoni, P., Ghisolfi, G., Negro, F. and Sapino, A. (1996) "Immunolocalization and gene expression of oxytocin receptors in carcinomas and non-neoplastic tissues of the breast", *Am. J. Pathol.* **148**, 1895–1903.

Elands, J., Resink, A. and De Kloet, E.R. (1990) "Neurohypophysial hormone receptors in the rat thymus, spleen and lymphocytes", *Endocrinology* **126**, 2703–2710.

Ericsson, A., Geenen, V., Robert, F., Legros, J.J., Vrindts-Gevaert, Y., Franchimont, P., Brene, S. and Persson, H. (1990) "Expression of preprotachykinin A and neuropeptide-Y messenger RNA in the thymus", *Mol. Endocrinol.* **4**, 1211–1218.

Gainer, H. and Wray, S. (1994) "Cellular and molecular biology of oxytocin and vasopressin", In: Knobil, E. and Neill, J.D., eds, The Physiology of Reproduction, 2nd Edn. (Raven Press, New York), pp 1099–1130.

Geenen, V., Kecha, O. and Martens, H. (1998) "Thymic expression of neuroendocrine self-peptides precursors: role in T-cell survival and self-tolerance", *J. Neuroendocrinol.* **10**, 811–822.

Geenen, V., Kecha, O., Brilot, F., Charlet-Renard, C. and Martens, H. (1999) "The thymic repertoire of neuroendocrine-related self antigens: biological role in T-cell selection and pharmacological implications", *NeuroImmunomodul.* **6**, 115–125.

Geenen, V., Brilot, F., Hansenne, I., Kecha-Kamoun, O., Martens, H., "Thymus and T cells", In: Adelman, G. and Smith, B., eds, Encyclopedia of Neuroscience, 3rd Edn. (Elsevier, New York), in press.

Goren, H.J., Okabe, T., Lederis, K. and Hollenberg, M.D. (1984) "Oxytocin-stimulated glucose oxydation by rat thymocytes", *Proc. West. Pharmacol. Soc.* **27**, 983–986.

Jenkinson, E.J. and Anderson, G. (1994) "Fetal thymic organ cultures", *Curr. Opin. Immunol.* **6**, 293–297.

Kanazawa, S., Ilic, D., Hashiyama, M., Noumura, T., Yamamoto, T., Suda, T. and Aizawa, S. (1996) "p59fyn-p125FAK cooperation in development of CD4 + CD8 + thymocytes", *Blood* **87**, 865–870.

Kecha, O., Brilot, F., Martens, H., Franchimont, N., Renard, C., Greimers, R., De Fresne, M.-P., Winkler, R. and Geenen, V. (2000) "Involvement of the thymic insulin-like growth factor axis in early T-cell development: a study using fetal thymic organ cultures", *Endocrinology* **143**, 1209–1217.

Lieberman, M., Declève, A., Ricciardi-Castagnoli, P., Boniver, J., Finn, O.J. and Kaplan, H.S. (1979) "Establishment, characterization and virus expression of cell lines derived from radiation- and virus-induced lymphomas of C57BL/Ka mice", *Int. J. Cancer* **24**, 168–177.

Martens, H., Robert, F., Legros, J.-J., Geenen, V. and Franchimont, P. (1992) "Expression of functional neurohypophysial peptide receptors by immature and cytotoxic T-cell lines", *Prog. NeuroEndocrin. Immunol.* **5**, 31–39.

Martens, H., Goxe, B. and Geenen, V. (1996) "The thymic repertoire of neuroendocrine-related self-antigens: implications in T cell life and death", *Immunol. Today* **17**, 312–317.

Martens, H., Kecha, O., Charlet-Renard, Ch., Defresne, M.-P. and Geenen, V. (1998) "Neurohypophysial peptides stimulate the phosphorylation of pre-T cell focal adhesion kinases", *Neuroendocrinology* **67**, 282–289.

Mason, D.Y. (2000) Seventh Workshop on Human Leucocyte Differentiation Antigens.

Pleau, J.-M., Esling, A., Bach, J.-F. and Dardenne, M. (1996) "Gene expression of pancreatic glutamic acid decarboxylase in the nonobese diabetic mouse", *Biochem. Biophys. Res. Commun.* **220**, 399–404.

Robert, F., Geenen, V., Schoenen, J., Burgeon, E., De Groote, D., De Fresne, M.-P., Legros, J.-J. and Franchimont, P. (1991) "Colocalization of immunoreactive oxytocin, vasopressin and interleukin-1 in human thymic epithelial neuroendocrine cells", *Brain Behav. Immun.* **5**, 102–115.

Robert, F., Martens, H., Cormann, N., Bendida, A., Schoenen, J. and Geenen, V. (1992) "The recognition of hypothalamo-neurohypophysial functions by developing T cells", *Dev. Immunol.* **2**, 131–140.

Sugimoto, T., Saito, M., Mochizuki, S., Watanabe, Y., Hashimoto, S. and Kawashima, H. (1994) "Molecular cloning and functional expression of cDNA encoding the human V1b vasopressin receptor", *J. Biol. Chem.* **269**, 27088–27092.

Thibonnier, M., Boyer, A.L. and Leng, Z. (1993) "Cytoplasmic and nuclear signalling pathways in V1 vascular vasopressin receptors", *Regul. Pept.* **45**, 79–84.

Torres, B.A. and Johnson, H.M. (1988) "Arginine vasopressin (AVP) replacement of helper cell requirement in IFN-$\gamma$ production. Evidence for a novel AVP receptor on mouse lymphocytes", *J. Immunol.* **140**, 2179–2183.

Vanneste, Y., Ntodou-Thome, A., Vandersmissen, E., Charlet, C., Franchimont, D., Martens, H., Lhiaubet, A.M., Schimpff, R.M.,

Rostène, W. and Geenen, V. (1997) "Identification of neurotensin-related peptides in human thymic epithelial cell membranes and relationship with major histocompatibility complex class I molecules", *J. Neuroimmunol.* **76**, 161–166.

Vink, A., Renauld, J.C., Warnier, G. and Van Snick, J. (1993) "IL-9 and mouse thymic lymphomas", *Eur. J. Immunol.* **23**, 1134–1138.

Whitfield, J.F., Perris, A.D. and Youdale, T. (1969) "The calcium-mediated promotion of mitotic activity in rat thymocyte populations by growth hormone, neurohormones, parathyroid hormone and prolactine", *J. Cell. Physiol.* **73**, 203–211.

Zingg, H.H. (1996) "Vasopressin and oxytocin receptors", *Bailliere's Clin. Endocr. Metab.* **10**, 75–96.

